# Side Effects to Systemic Glucocorticoid Therapy in Dogs Under Primary Veterinary Care in the UK

**DOI:** 10.3389/fvets.2020.00515

**Published:** 2020-08-14

**Authors:** Doaa A. Elkholly, Dave C. Brodbelt, David B. Church, Ludo Pelligand, Kennedy Mwacalimba, Andrea K. Wright, Dan G. O'Neill

**Affiliations:** ^1^Royal Veterinary College, Pathobiology and Population Health, London University, London, United Kingdom; ^2^Royal Veterinary College, Clinical Science and Services, London University, London, United Kingdom; ^3^Royal Veterinary College, Comparative Biomedical Science, London University, London, United Kingdom; ^4^Zoetis, Outcomes Research, Parsippany, NJ, United States; ^5^Zoetis, Outcomes Research, Dublin, Ireland

**Keywords:** glucocorticoids, side effects, dogs, veterinary care, VetCompass, corticosteroid, polyuria, polydipsia

## Abstract

**Objectives:** Systemic glucocorticoids are widely used in companion animals. This study aimed to estimate the frequency, describe the characteristics and to evaluate risk factors for common side effects to systemic glucocorticoid therapy in dogs under primary veterinary care in the UK.

**Methods:** A cohort study using VetCompass™ data from 455,557 dogs under primary veterinary care during 2013 estimated the frequency of side effects to systemic glucocorticoid therapy occurring within 31 days of therapy. Risk factors for the most common side effects, polyuria and polydipsia (PUPD), were evaluated using multivariable logistic regression modeling (*P* < 0.05).

**Results:** During 2013, 28,472 study dogs received systemic glucocorticoids (6.2%, 95% CI 6.2–6.3). Review of the records of 3,000 randomly selected treated dogs identified 148 (4.9%, 95% CI 4.2–5.7%) dogs with at least one side effect recorded within 31 days of therapy. The most frequent side effects were polydipsia (39.2% of total presenting signs), polyuria (28.4%), vomiting (16.2%) and diarrhea (14.9%), dogs receiving only oral systemic glucocorticoids (odds ratio, OR: 3.72) and dogs receiving both oral and injectable systemic glucocorticoid (OR: 10.71) had increased odds of PUPD compared with dogs receiving only injectable systemic glucocorticoid. Focusing on the active substance used, treatment with prednisolone tablets only (OR: 3.53) and treatment with both prednisolone tablets and injectable dexamethasone sodium phosphate (OR: 7.62) showed increased odds of PUPD compared to treatment with injectable dexamethasone sodium phosphate only.

**Brief:** These results can assist veterinarians to optimize therapeutic selection for reduced side effect, to inform owners on common side effects, and help protect the welfare of pets and their owners.

## Introduction

Glucocorticoids are commonly used in companion animal veterinary practice as anti-inflammatory and immunosuppressive agents (1, 2). However, glucocorticoids have been associated with various side effects, including vomiting, diarrhea, bodyweight gain or loss, polyuria, polydipsia, delayed wound healing, behavioral problems, immunosuppression and predisposition to infection (3, 4).

There is some published evidence on side effects that may occur following systemic glucocorticoid usage. Systemic glucocorticoid treatment has been associated with predisposition to infection (5, 6) immunosuppression and decreased urine osmolality (1, 2, 7). Glucocorticoid treatment has been associated with increased risk of urinary tract infection among dogs with skin disease (5, 6, 8). Dogs administered long-term hydrocortisone showed side effects including thinning of skin, PUPD (9). Another study reported increased surgical complications, diarrhea and urinary tract infection in dogs with acute thoracolumbar intervertebral disk herniation treated with dexamethasone before surgery compared with dogs treated with other glucocorticoids (10). Dogs treated with dexamethasone and methylprednisolone acetate have also been reported to have an increased risk of hepatopathy (11). Despite this qualitative evidence, there is little published quantitative evidence on the frequency or risk factors for side effects following systemic glucocorticoids therapy.

Anonymised clinical data from primary-care veterinary practices offers potential to investigate the frequency of side effects following glucocorticoid therapy (12). Previous studies within the VetCompass™ Programme used data collected from primary-care small animal practices to quantify the usage of glucocorticoids in dogs but did not report on side effects (13, 14). The current study aimed to estimate the frequency and describe the characteristics of side effects to systemic glucocorticoid therapy recorded in the electronic clinical records from a large population of dogs under primary veterinary care in the UK. The study further aimed to evaluate risk factors for PUPD as side effects in dogs treated with systemic glucocorticoids.

## Methods

The VetCompass Programme collates de-identified electronic patient record (EPR) data from primary-care veterinary practices in the UK (15). VetCompass collects information fields with relevant dates that include species, breed, date of birth, sex, neuter status, insurance status, bodyweight, clinical notes and treatment. The EPR data were extracted from practice management systems using integrated clinical queries and uploaded to the VetCompass database (16).

A retrospective cohort study of dogs attending VetCompass practices was used to estimate the frequency and risk factors for PUPD after systemic glucocorticoid therapy. The sampling frame for the study included dogs under veterinary care during 2013 that had at least one EPR within the VetCompass database from January 1st 2013 to December 31st 2013 and/or at least one EPR both before and after this period. Sample size calculations estimated that ~3,000 dogs would be required to be studied in order to detect a 0.5% side effects frequency (with a precision of ± 0.25% confidence limit and 95% confidence level). Ethical approval was granted by the RVC Ethics and Welfare Committee (URN 2015 1369).

A systemic glucocorticoid treatment was defined as any glucocorticoid preparation administered by injection, dispensed as oral tablets or an oral suspension. Inclusion criteria required that a systemic glucocorticoid was either administered or dispensed during 2013. Animals receiving only topical glucocorticoid therapy were excluded from consideration. A comprehensive list of chemical and brand names for systemic glucocorticoids was generated by searching the Veterinary Medicines Directorate (17), British Small Animal Veterinary Association (BSAVA) formulary (18) and National Office for Animal Heath (NOAH) veterinary products databases (19). A list of search terms was derived from these chemical and brand names to identify all systemic glucocorticoids within the treatment field of the VetCompass database. Case-finding involved initial screening of the treatment fields of all dogs within the study population for any treatment record that may have been a systemic glucocorticoid based on these search terms. A list of animal unique identification codes for these candidate cases for systemic glucocorticoid usage was generated and treatment data (date, drug, volume of injectable/number of tablets, dosage instructions) on all treatments during 2013 were extracted from the VetCompass database to an Excel format. Duplicates were removed and all remaining dispensing terms used were manually coded as systemic glucocorticoid (yes/no) to create a master list of systemic glucocorticoid dispensing terms. Data were extracted to describe the name of the active substance, dosage and route of administration of glucocorticoids used.

For the current study, a side effect case was defined as a dog with evidence in the clinical notes of a side effect within 31 days of a new episode of systemic glucocorticoid usage during 2013. This period was selected to allow for manifestation of potentially delayed onset side-effects whilst reducing the inclusion of potential side-effect events unrelated to glucocorticoid therapy. It was required that there was no evidence in the clinical notes of the observed potential side effect(s) in the 31 days prior to the new systemic glucocorticoid episode. A new episode was defined as systemic glucocorticoid usage that was not preceded by systemic glucocorticoid therapy during the previous 31 days. Individual treatment episodes could include the usage of multiple systemic glucocorticoid products and dogs could have multiple treatment episodes during 2013. All dogs that received at least one systemic glucocorticoid during 2013 were randomly ordered and the EPRs of a random sample of 3,000 of these dogs were manually reviewed in detail to identify all dogs that met the side effects case definition. Data were extracted for identified side effects cases to describe the dates of the start of the episode of systemic glucocorticoid usage and the first reported side effect, the clinical sign(s) of the side effect, the clinical action(s) taken in response to the side effect and the main clinical indication for the systemic glucocorticoid originally prescribed in the side effects cases.

The period (days) of glucocorticoid administration prior to the side effect was calculated from the glucocorticoid episode start date to the first reported side effect. Initial duration of systemic glucocorticoid prescribed for side effects cases was extracted from the treatment data field. The initial daily systemic glucocorticoid dosage (mg/kg/day) was calculated from the total daily dose for each glucocorticoid from the treatment data field and dividing by the bodyweight.

A *breed* variable included individual breeds with 60 or more systemic glucocorticoid cases in the random sample of 3,000 dogs, a grouped category of all remaining purebreds and a general grouping of crossbred dogs. This approach was taken to allow focus on commonly affected breeds and to facilitate statistical power for the individual breed analyses (20). A *purebred* variable categorized all dogs of recognizable breeds as “purebred” and the remaining dogs as “crossbred” (21). Age (years) was calculated for side effect cases at the date of first administration of the systemic glucocorticoid episode for the relevant side effects and for non-case dogs at the date of administration of a randomly selected systemic glucocorticoid event during 2013. An *age* variable categorized age (years) into four groups (≤ 2.0, > 2.0 to ≤ 5.0, > 5.0 to ≤ 8.0, > 8.0 years). Bodyweight (kg) described the nearest bodyweight recorded during 2013 to the date of the systemic glucocorticoid usage episode for dogs ≥ 18 months and the nearest bodyweight recorded within 1 month to the systemic glucocorticoid usage episode date for dogs ≤ 18 month. A *bodyweight* variable categorized bodyweight into six groups (0.0–10.0, > 10.0–20, > 20.0–30, > 30.0–40 kg, ≥ 40.0 kg, unavailable). *Route of administration* and *active substance* variables were defined for side effects cases on all systemic glucocorticoid products used within a maximum of 31 days from the start of the systemic glucocorticoid episode up to the onset of a side effect. For non-case dogs, these data were extracted for treatment within 31 days of the start of a systemic glucocorticoid episode that was randomly selected from all systemic glucocorticoid episodes for that dog. A *season* variable defined the season of treatment based on the age date defined above and categorized into winter (December to February), summer (June to August), autumn (September to November) and spring (March to May).

Following data checking and cleaning in Excel (Microsoft Office Excel 2013, Microsoft Corp.), analyses were conducted using Stata Version 14 (Stata Corporation). Numerical data were assessed graphically for normality and summarized with median (interquartile range, IQR) or mean (standard deviation) as appropriate (22). Risk factor analysis evaluated the 3,000 randomly selected dogs treated with at least one systemic glucocorticoid to compare PUPD cases with the non- PUPD dogs. These two side effects were considered closely related therefore any dog with at least one of these PUPD side effects was included in the study. Logistic regression modeling was used to evaluate univariable associations between risk factors and PUPD outcome. Risk factors with liberal associations in univariable modeling (*P* < 0.3) were taken forward for mixed effects multivariable logistic regression modeling using a manual backwards-stepwise elimination method. Residual interclass correlation coefficients were assessed, clinic was evaluated as a random effect and pair-wise interaction effects were evaluated for the final model variables. Two multivariable models were developed to alternatively evaluate two strongly biologically correlated variables of interest separately: route of administration of the steroids and active substances used. The Hosmer-Lemeshow test for goodness of fit was conducted for assessment of the fit of the final fixed effects multivariable model (23). Final statistical significance was set at *P* < 0.05.

## Results

The study population included 455,557 dogs from 304 clinics in the VetCompass database under veterinary care during 2013. Of these, 28,472 dogs received at least one systemic glucocorticoid during 2013 giving an overall one-year period prevalence for systemic glucocorticoid usage of 6.2% (95% CI 6.2–6.3). From a random sample of 3,000 dogs receiving systemic glucocorticoids during 2013 that were reviewed in detail, 148 dogs were identified with at least one side effect within 31 days of administration, giving a side effects occurrence among dogs receiving systemic glucocorticoids of 4.9% (95% CI 4.2–5.7%). Of these 148 side effects reported, 97 (65%) occurred within the first 14 days after initiation of therapy. None of the side effects cases had more than one event of side effect during 2013.

The most common clinical categories leading to prescribing of systemic glucocorticoid to the 148 side effects cases were skin conditions (*n* = 50, 32.5%), ear problems (33, 21.4%) and gastro-intestinal tract problems (20, 13.0%) (Table 1). The most common clinical indications were dermatitis (38, 24.7%) and ear infections (28, 18.2%). Prednisolone 5 mg was the most common oral systemic glucocorticoid prescribed for dogs with a side effect *without a preceding systemic glucocorticoid injection*, with a median initial daily dosage of 0.67 mg/kg/day (Table 2). Similarly, prednisolone 5 mg was the most common oral systemic glucocorticoid prescribed for side effect cases *with a preceding systemic glucocorticoid injection* (median initial daily dosage of 0.48 mg/kg/day). Dexamethasone sodium phosphate was the most common injectable systemic glucocorticoid used in side effects cases (median initial daily dosage 0.10 mg/kg/day). The duration of dispensed treatments for side effects cases varied with active substance and formulation type though was generally for a median duration of ~2–3 weeks (Table 3).

**Table 1:** Main clinical indications for systemic glucocorticoid usage in 148 side effects cases recorded within 31 days after receiving systemic glucocorticoid therapy among dogs under primary veterinary care in the UK. Some cases had multiple indications.

**Clinical category**	**Main clinical indication**	**No. side effects (%)**	**Total (%)**
Skin	Dermatitis	38 (24.7)	50 (32.5)
	Pyoderma	5 (3.2)	
	Insect bite	2 (1.3)	
	Skin problem (not specified and other)	5 (3.2)	
Otic problem	otitis	28 (18.2)	29 (18.8)
	Ear lipoma	1 (0.6)	
Gastro-intestinal tract	Diarrhea	8 (5.2)	20 (13)
	Vomiting	4 (2.6)	
	Vomiting and gastritis	1 (0.6)	
	Vomiting and dermatitis	1 (0.6)	
	Constipation	1 (0.6)	
	Inflammation bowel disease	2 (1.3)	
	Stomach ulcer	1 (0.6)	
	Gastrointestinal problem (not specified)	2 (1.3)	
Neoplasia	Abdominal tumor	2 (1.3)	13 (8.4)
	Lung neoplasia	3 (1.9)	
	Prostatic carcinoma	2 (1.3)	
	Mast cell tumor	1 (0.6)	
	Oral and pharyngeal tumors	2 (1.3)	
	Mammary adenocarcinoma	1 (0.6)	
	Cancer (not specified)	2 (1.3)	
Respiratory	Coughing	3 (1.9)	11 (7.1)
	Bacterial pneumonia	1 (0.6)	
	Lung fibrosis	1 (0.6)	
	Lung consolidation	1 (0.6)	
	Brachycephalic Obstructive Airway Syndrome (BOAS)	1 (0.6)	
	Bronchitis	1 (0.6)	
	Coughing and ventral oedema,	1 (0.6)	
	Kennel cough	1 (0.6)	
	Rhinitis due to allergy	1 (0.6)	
Other	After surgery (hemimandibulectomy to remove tumor)	2 (1.3)	11 (7.1)
	Allergy	2 (1.3)	
	Anal gland inflammation	4 (2.6)	
	Cyst in limb	1 (0.6)	
	Lethargic	1 (0.6)	
	Prostate problem	1 (0.6)	
Nervous system	CNS inflammatory diseases	2 (1.3)	10 (6.5)
	Spinal impingement	2 (1.3)	
	Tremors (cause not specified)	1 (0.6)	
	Seizure	1 (0.6)	
	Vestibular syndrome	4 (2.6)	
Autoimmune	Autoimmune myositis	1 (0.6)	4 (2.6)
	Immune mediated anemia	2 (1.3)	
	Immune-mediated thrombocytopenia	1 (0.6)	
Skeletal	Arthritis	3 (1.9)	4 (2.6)
	Mandible bony swelling	1 (0.6)	
Adrenal	Addison's disease	2 (1.3)	2 (1.3)
Total			154

**Table 2:** Initial dosages of systemic glucocorticoid active substances that had a side effect recorded within 31 days after receiving systemic glucocorticoid therapy among dogs under primary veterinary care in the UK (*n* = 120).

**Active substance**	**No. dogs**	**Median**	**IQR**	**Range**	**Expected duration (injectable)**
**Oral tablets without injection (mg/kg daily)**					
Prednisolone 1 mg	2 (1.4)	0.14	0.11–0.17	0.11–0.17	
Prednisolone 5 mg	58 (39.2)	0.67	0.44–0.92	0.20–2.22	
Prednisolone 25 mg	12 (8.1)	0.89	0.65–1.29	0.40–2.50	
Methylprednisolone	4 (2.7)	0.97	0.63–1.94	0.47–2.74	
Prednisolone/cinchophen	1 (0.7)	0.12			
Overall tablets without injection (mg/kg daily)	77 (52.0)	0.7	0.46–1.0	0.11–2.74	
**Tablets with prior injection (mg/kg daily)**					
Prednisolone 5 mg	6 (4.1)	0.48	0.37–0.54	0.20–0.75	
Prednisolone 25 mg	1 (0.7)	0.89			
Overall tablets with prior injection (mg/kg daily)	7 (4.7)	0.52	0.37–0.75	0.20–0.89	
**Injectable (mg/kg daily)**					
Dexamethasone isonicotinate	3 (2.0)	0.10	0.07–0.11	0.07–0.11	Intermediate
Dexamethasone phenylpropionate, dexamethasone sodium phosphate	9 (6.1)	0.07	0.06–0.09	0.05–0.20	Short to intermediate
Dexamethasone sodium phosphate	23 (15.5)	0.10	0.09–0.17	0.07–0.86	Short
Methylprednisolone acetate	1 (0.7)	2.27			Long
Overall injectables (mg/kg daily)	36 (24.3)	0.10	0.08–0.15	0.05–2.27	
Total	120				

*Twenty-eight cases were missing bodyweight data so the daily dosage was not available*.

**Table 3:** Duration of systemic glucocorticoid active substances dispensed for oral treatments that had a side effect recorded within 31 days after receiving systemic glucocorticoid therapy among dogs under primary veterinary care in the UK (*n* = 148).

**Active substance**	**No. dogs (% of side effect cases)**	**Median (days)**	**IQR**	**Range (days)**
**Oral tablets without preceding injection**				
Prednisolone 1 mg	4 (2.7)	22.5	14.5–60	14–90
Prednisolone 5 mg	61 (41.2)	15.0	12–30	4–180
Prednisolone 25 mg	15 (10.1)	14.0	10–28	7–120
Methylprednisolone	4 (2.7)	36.5	19–59	14–69
Prednisolone/cinchophen	1 (0.7)	10		
Overall tablets without preceding injection	85 (57.4)	15.0	12–30	4–180
**Oral tablets with preceding injection**				
Prednisolone 5 mg	12 (8.1)	10.0	6.0–16.5	4–31
Prednisolone 25 mg	2 (1.4)	21.0	20–22	20–22
Overall tablets with preceding injection	14 (9.5)	11.5	7.0–20	4–31

The period to onset of the side effects varied across side effect cases. For prednisolone administrations without preceding injection, the median duration between start of glucocorticoid treatment and the development of side effects varied from 10.5 to 13 days, whilst methylprednisolone had a median onset of 4.5 days, though this was based on only 4 events (Table 4). Dexamethasone sodium phosphate, the most commonly administered injectable systemic glucocorticoid, had a median onset to side effects of 7 days based on a single injection course for all these events.

**Table 4:** Period (days) from commencing systemic glucocorticoid treatment to developing a side effect for dogs under primary veterinary care in the UK (*n* = 148).

**Active substance**	**No. dogs (%)**	**Median (days)**	**IQR**	**Range (days)**
**Oral tablets without preceding injection**				
Prednisolone 1 mg	4 (2.7)	11	5.0–15.5	3.0–16
Prednisolone 5mg	65 (43.9)	13	7.0–18.0	1.0–30
Prednisolone 25mg	16 (10.8)	10.5	6.5–19.0	4.0–25
Methylprednisolone	4 (2.7)	4.5	1.0–13.5	0–20
Prednisolone/cinchophen	1 (0.7)	5		
Overall tablets without preceding injection	90 (60.8)	12.5	7.0–18.0	0–30
**Tablets with preceding injection**				
Prednisolone 5 mg	12 (8.1)	9	6.0–9.5	3.0–20
Prednisolone 25 mg	2 (1.4)	1.5	0–3.0	0–3.0
Overall tablets with preceding injection	14 (9.5)	8	3.0–9.0	0–20
**Injectable only**				
Dexamethasone Isonicotinate	3 (2.0)	7	6.0–21	6.0–21
Dexamethasone phenylpropionate, dexamethasone sodium phosphate	11 (7.4)	17	11–24	2.0–28
Dexamethasone sodium phosphate	29 (19.6)	7	4.0–14	1.0–31
Methylprednisolone Acetate	1 (0.7)	6		
Overall injectables	44 (29.7)	8	5.5–20	1.0–31
Total	148			

There were 241 clinical signs recorded in the EPR within the 148 side effects cases (Table 5). Some side effects cases had more than one clinical sign recorded. No fatality was reported. The most frequent clinical signs reported for side effects cases were polydipsia (58 events, 39.2% of the side effects cases), polyuria (42, 28.4%), vomiting (24, 16.2%), diarrhea (22, 14.9%) and polyphagia (20, 13.5%). The dose of the systemic glucocorticoid was subsequently reduced in 43 (29.1%) side effects cases whilst therapy was discontinued in 25 (16.9%) side effects cases.

**Table 5:** Frequency (%) of presenting signs among 148 dogs under primary veterinary care with side effects recorded within 31 days after receiving systemic glucocorticoid therapy.

**Side effect**	**Frequency**	**% of side effects dogs**
Polydipsia	58	39.2
Polyuria	42	28.4
Vomiting	24	16.2
Diarrhea	22	14.9
Polyphagia or increased appetite	20	13.5
Weight gain	18	12.2
Weight loss	14	9.5
Anorexia	11	7.4
Lethargy	10	6.8
Panting	8	5.4
Hair loss or alopecia	3	2.0
Behavioral change	3	2.0
Growling	3	2.0
Shaking	3	2.0
Mastitis	1	0.7
Delayed wound healing	1	0.7
Total	241	

*Some dogs had more than one presenting sign recorded*.

Seventy-one dogs had either PUPD. Of these, 53 (75.71%) were purebred and 40 (56.34%) were male. PUPD cases had a median bodyweight of 19.1 kg (IQR 10.8–30.4) and median age was 6.9 years (IQR 4.2–10.2), (Table 6). The most common breeds among the PUPD cases were Labrador Retriever (8/71, 11.27%), West Highland White Terrier (4/71, 5.63%), Boxer (4/71, 5.63%), along with crossbred dogs (17/71, 23.94%) (Table 6). Of the non-cases dogs, 2266 (77.55%) were purebred, 1630 (55.65%) were male. The median bodyweight for non-cases was 14.3 kg (IQR 8.6–25.0) and the median age was 4.9 years (IQR 2.1–8.3). The most common breeds among the non-case dogs were Staffordshire Bull Terrier 23 0 (7.85%), Labrador Retriever (179, 6.11%), Jack Russell Terrier (178, 6.08%) as well as crossbreds 593 (20.25%).

**Table 6:** Descriptive analysis and univariable logistic regression results for risk factors for polyuria and/or polydipsia within 31 days after receiving systemic glucocorticoid therapy among dogs under primary veterinary care in the UK.

**Variable**	**Category**	**Case No. (%)**	**Non-case No. (%)**	**Odds ratio**	**95% CI**	**Category *P*-value**	**Variable *P-*value**
Purebred status	Crossbred	17 (24.29)	656 (22.45)	Base			0.719
	Purebred	53 (75.71)	2266 (77.55)	0.9	0.52–1.57	0.716	
Common breeds	Crossbreed	17 (23.94)	593 (20.25)	Base			0.39
	Boxer	4 (5.63)	61 (2.08)	2.29	0.75–7.01	0.148	
	Bichon	3 (4.23)	67 (2.29)	1.56	0.45–5.47	0.486	
	Labrador Retriever	8 (11.27)	179 (6.11)	1.56	0.66–3.67	0.31	
	German Shepherd Dog	3 (4.23)	71 (2.42)	1.47	0.42–5.15	0.544	
	Lhasa Apso	2 (2.82)	59 (2.01)	1.18	0.27–5.24	0.825	
	Border Collie	2 (2.82)	62 (2.12)	1.13	0.25–4.98	0.877	
	Cavalier King Charles Spaniel	0	68 (2.32)	Empty			
	Cocker Spaniel	3 (4.23)	111 (3.79)	0.94	0.27–3.27	0.926	
	West Highland White Terrier	4 (5.63)	171 (5.84	0.82	0.27–2.46	0.718	
	Shih Tzu	2 (2.82)	97 (3.31)	0.72	0.16–3.16	0.663	
	Yorkshire Terrier	2 (2.82)	105 (3.58)	0.66	0.15–2.92	0.588	
	Staffordshire Bull Terrier	3 (4.23)	230 (7.85)	0.45	0.13–1.57	0.212	
	Jack Russell Terrier	1 (1.41)	178 (6.08)	0.20	0.03–1.48	0.114	
	Other purebred	17 (23.94)	877 (29.94)	0.68	0.34–1.34	0.26	
Sex	Female	31 (43.66)	1295 (44.21)	Base			0.918
	Male	40 (56.34)	1630 (55.65)	1.03	0.64–1.65	0.918	
Age (years)^a^	≤2.0	5 (7.04)	713 (24.47)	Base			<0.001
	2.0– ≤ 5.0	20 (28.17)	808 (27.73)	3.53	1.32–9.45	0.012	
	5.0– ≤ 8.0	17 (23.94)	615 (21.11)	3.94	1.45–10.75	0.007	
	>8.0	29 (40.85)	778 (26.70)	5.32	2.05–13.81	0.001	
Bodyweight (kg)^b^	≤10.0	12 (16.90)	810 (27.65)	Base			0.319
	>10.0–20	17 (23.94)	739 (25.23)	1.55	0.74–3.27	0.247	
	>20.0–30	13 (18.31)	429 (14.65)	2.05	0.93–4.52	0.077	
	>30.0–40	9 (12.68)	266 (9.08)	2.28	0.95–5.48	0.064	
	>40	5 (7.04)	134 (4.57)	2.52	0.87–7.26	0.087	
	Not recorded	15 (21.13)	551 (18.81)	1.84	0.85–3.96	0.120	
Route of systemic glucocorticoid administration (within 31 days)	Injectable	10 (14.08)	1,353 (46.19)	Base			<0.001
	Oral	42 (59.15)	1,349 (46.06)	4.21	2.10–8.43	<0.001	
	Both oral and injectable	19 (26.76)	227 (7.75)	11.32	5.20–24.67	<0.001	
Active systemic glucocorticoid substance (Within 31 days)	Injectable: Dexamethasone Sodium Phosphate	7 (9.86)	805 (27.48)	Base			<0.001
	Injectable: Dexamethasone Phenylpropionate, Dexamethasone Sodium Phosphate	2 (2.82)	457 (15.60)	0.5	0.10–2.43	0.393	
	Other injectable only	2 (2.82)	92 (3.14)	2.5	0.51–12.21	0.258	
	Oral: Methylprednisolone	0	120 (4.10)	Empty			
	Oral: Prednisolone	44 (61.97)	1,233 (42.10)	4.1	1.84–9.16	<0.001	
	Oral: Prednisolone- and injectable Dexamethasone Sodium Phosphate	13 (18.31)	184 (6.28)	8.12	3.20–20.65	<0.001	
	Other oral and injectable combinations	3 (4.23)	38 (1.30)	9.08	2.26–36.49	<0.001	
Season	Winter	14 (19.72)	621 (21.20)	Base			0.075
	Spring	24 (33.80)	609 (20.79)	1.75	0.9–3.41	0.102	
	Autumn	17 (23.94)	780 (26.63)	0.97	0.47–1.98	0.926	
	Summer	16 (22.54)	919 (31.38)	0.77	0.37–1.59	0.484	
Neuter status	Entire	13 (18.31)	981 (33.49)				0.018
	Neutered	50 (70.42)	1,671 (57.05)	2.26	1.22–4.18	0.009	
	Unrecorded	8 (11.27)	277 (9.46)	2.18	0.89–5.31	0.087	

a *Age at having a side effect for cases and age at a random treatment for non-cases*.

b *Within 31 days of treatment for dogs <18 month and any body weight in 2013 for adults*.

Univariable logistic regression identified four of nine tested variables that were liberally associated with PUPD and were further evaluated using multivariable logistic regression modeling: age, route of administration, active substance and neuter statu*s*. Add in the ones that were dropped also. There is strong biological correlation between route of administration and active substance so these were evaluated in separate multivariable modeling that resulted in two final models.

The final model that focused on route of administration retained 2 variables: *route of administration* and *age*. After accounting for the effects of the age, dogs that had both oral and injectable systemic glucocorticoids (OR: 10.40 95% CI 4.76–22.75) and dogs that had oral systemic glucocorticoids (OR: 3.69 95% CI 1.84–7.42) showed increased odds of PUPD compared with dogs that had injectable systemic glucocorticoids only (Table 7). Dogs aged > 8 years had 4.24 times the odds (95% CI 1.62–11.09) of PUPD compared with dogs aged ≤ 2 years. No biologically significant interactions were identified. Clinic was not significant as a random effect (*P*-value = 0.252). There was no evidence of poor fit in the final fixed effect multivariable model (Hosmer-Lemeshow *P*-value = 0.789).

**Table 7:** Final model 1: Multivariable logistic regression results with route of administration as the focus for risk factors for polyuria and polydipsia within 31 days after receiving systemic glucocorticoid therapy among dogs under primary veterinary care in the UK.

**Variable**	**Category**	**Odds ratio**	**95% CI**	**Category *P-value***	**Variable *P-value***
Route of administration (within 31 days)	Injectable	Base			<0.001
	Oral	3.69	1.84–7.42	<0.001	
	Both oral and injectable	10.4	4.76–22.75	<0.001	
Age (years)	≤2.0	Base			0.005
	2.1–5.0	3.13	1.16–8.44	0.024	
	5.1–8.0	3.23	1.18–8.86	0.023	
	>8.0	4.24	1.62–11.09	0.003	

The final model that focused on active substance also retained two variables: active substance and age. Dogs treated with prednisolone tablets only had 3.44 (95% CI 1.54–7.73) times the odds of a PUPD compared with dogs that had injectable dexamethasone sodium phosphate only (Table 8). Dogs treated with both prednisolone tablets and injectable dexamethasone sodium phosphate had 7.29 (95% CI 2.86–18.60) times the odds of PUPD compared with dogs that had injectable dexamethasone sodium phosphate alone. Dogs aged > 8 years had 4.38 times the odds (95% CI 1.67–11.47) of PUPD compared with dogs aged ≤ 2 years. Clinic was not significant as a random effect (*P*-value = 0.198). No biologically significant interactions were identified and there was no evidence of poor fit in the final fixed effect multivariable model (Hosmer-Lemeshow *P*-value = 0.579).

**Table 8:** Final model 2: Multivariable logistic regression results with active substance as the focus for risk factors for polyuria and polydipsia within 31 days after receiving systemic glucocorticoid therapy among dogs under primary veterinary care in the UK.

**Variable**	**Category**	**Odds ratio**	**95% CI**	**Category *P-value***	**Variable *P-value***
Active substance (within 31 days)	Injectable: Dexamethasone Sodium Phosphate	Base			<0.001
	Injectable: Dexamethasone Phenylpropionate, Dexamethasone Sodium Phosphate	0.45	0.09–2.19	0.325	
	Other injectable only	2.15	0.44–10.58	0.345	
	Oral: Methylprednisolone	Empty			
	Oral: Prednisolone	3.44	1.54–7.73	0.003	
	Oral and injectable: Prednisolone-Dexamethasone Sodium Phosphate	7.29	2.86–18.60	<0.001	
	Other oral and injectable	7.13	1.76–28.92	0.006	
Age (years)	≤ 2.0	Base			
	2.1–5.0	3.17	1.17–8.54	0.023	0.004
	5.1–8.0	3.38	1.23–9.29	0.018	
	>8.0	4.38	1.67–11.47	0.003	

## Discussion

This is the first study to explore side effects to systemic glucocorticoids in dogs attending primary-care practices by analyzing clinical records from a multi-center primary-care research database. The study identified that nearly 5% of dogs receiving a systemic glucocorticoid had evidence of a side effect recorded in the EPR within 31 days of onset of therapy.

Of the 148 side effects cases identified, none had more than one side effect event during 2013, suggesting that veterinarians may have taken steps to avoid recurrent side effect events to systemic glucocorticoids in known susceptible animals. Immediate clinical actions in response to side effects identified in the current study, included reduction of the glucocorticoid dose (29.1% of side effect events) and discontinuation of therapy (16.9% of side effect events). For consistency of the study, cases were only evaluated for the first course where side-effects were reported, though it would have been interesting to evaluate the number of these dogs that had subsequent courses within the study period.

Skin (32.5%) and ear (21.4%) conditions were the most common indications for prescribing systemic glucocorticoids where a side effect was subsequently observed. However, these findings are not conclusive evidence for a side effect predisposition by condition because the clinical indication for the non-side effect dogs was not studied, but it does suggest that this may be a useful future line of research. Many skin and ear disorders have an inflammatory or immune-mediated component that may benefit from systemic glucocorticoid therapy and consequently systemic glucocorticoids are routinely recommended for these conditions by dermatologists (24). A study using primary-care practice data in the UK reported that 20% of dermatology cases were prescribed systemic glucocorticoid therapy (25).

Prednisolone was the most commonly prescribed oral systemic glucocorticoid among the side effects cases. A previous study for the whole population of dogs under primary veterinary care in 2013 using VetCompass data reported similar results (14). The median prednisolone initial daily dosage was between 0.14 and 0.89 mg/kg/day, depending on tablet size. These were within the recommended manufacturer dosages of 0.1–2.0 mg/kg/day (2017). The short-acting agent, dexamethasone sodium phosphate, was the most commonly administered injectable systemic glucocorticoids among the side effects cases with a median daily dose of 0.10 mg/kg/day. Other injectable glucocorticoids, dexamethasone isonicotinate, dexamethasone phenylpropionate with dexamethasone sodium phosphate and methylprednisolone acetate were also used and are considered intermediate, short to intermediate and long acting respectively, so are less suitable for initiation of a combination therapy “injectable followed by oral treatment” plan. However, just under 5% of side effects occurred after injectable combined with oral glucocorticoid treatment were administered.

The median time to side effect after initiation of oral glucocorticoid therapy alone was 12.5 days, whilst side effects appeared to occur sooner when an injectable agent was administered either alone or with oral therapy, at a median of 8 days after onset of therapy. Injectable glucocorticoid therapy is likely to reach a higher plasma concentration sooner and hence any side effects might be expected to become evident sooner (3). However, there was a wide range in onset across agents and this reflects the variable nature of the side effects reported, such that certain side effects may occur more immediately after administration of the systemic glucocorticoids (e.g., PUPD) whilst others require prolonged exposure to occur (e.g., hair loss/alopecia) (26).

The two most frequent side effects to systemic glucocorticoids recorded in the current study were polydipsia (39.2%) and polyuria (28.4%). For this reason, factors associated with these side effects were explored more deeply in the analysis. These side effects likely result from the effect of glucocorticoids on the antidiuretic hormone (ADH) leading to excessive loss of fluids in urine and enhancing feeling of thirst to replace fluid loss (26). Polydipsia and polyuria can promote urinary incontinence by increasing bladder fill beyond the physiological thresholds of the urinary sphincter (27). Urinary incontinence has been considered to negatively impact on the welfare of affected dogs (28). Within the 31 day period PUPD were frequently reported however, urinary incontinence was not observed in the records.

The next most frequent side effect in the current study were vomiting (16.2%) and diarrhea (14.9%). Vomiting is associated with unpleasant nausea sensations, while profuse or prolonged vomiting can cause dehydration, electrolyte and acid-base imbalances that may require hospitalization (29, 30). Many human surgical patients report nausea and vomiting to be more distressing than the postsurgical pain (31). Behavioral changes (3 cases) and growling (3 cases) contributed 4% to the side effects cases in the current study. Previous study found an association between corticosteroids therapy and dog behavioral problems (4). In this study aggression toward people was the most recorded behavioral side effect. Therefore, as well as side effects to systemic glucocorticoids being relatively common, the results of the current study suggest that the clinical signs induced by these side effects can have substantial negative impacts for both owners and their dogs.

Dogs over 8 years old were more than 4 times more likely to have PUPD following systemic glucocorticoid treatment compared to dogs aged under 2 years. Increasing predisposition to incontinence (32) and chronic kidney disease (27) as dogs age may promote increased PUPD in older dogs. An experimental study reported higher liver enzymes and pathological hepatic changes in older dogs after glucocorticoids therapy compared to young dogs (33). Overall, these findings suggest that veterinarians should be especially vigilant about prescribing systemic glucocorticoids to older dogs. Sex did not appear to be associated with side-effects in the current study. A previous study that reported increased susceptibility of acquired urinary incontinence in neutered bitches compared to male dogs and this may suggest increased risk of PUPD in female dogs (28). However, the small number of cases may have underpowered the current study in identifying this association.

Dogs treated with oral systemic glucocorticoids had 3.69 times the odds, and dogs treated with both oral and injectable systemic glucocorticoids had 10.40 times the odds, of PUPD compared to dogs treated only with an injectable systemic glucocorticoid. Similarly, dogs receiving oral prednisolone alone had 3.44 times the odds, and dogs receiving a combination of oral prednisolone with injectable dexamethasone sodium phosphate had 7.29 times the odds of PUPD than dogs that received injectable dexamethasone sodium phosphate alone. The current results suggest prolonged and combination therapy compared to single injectable glucocorticoid administration may result in increased probability of PUPD. In humans, oral therapies are associated with prolonged exposure and maintenance of blood levels of these drugs and therefore suggest that the cumulative effect over time may be a strong contributor to PUPD risk (32, 34).

The data resource used for this study had some limitations as previously reported (13, 35). These EPR data were not recorded primarily for research purposes and thus were limited by reliance on accurate and thorough record-keeping by the clinicians. It was assumed all side effects would be recorded by the attending veterinarians, though it is possible that common side effects may not have been recorded, due to the anticipation of such side effects, suggesting that the current results may underestimate the frequency of complications. Owners' previous experience of expected side effects from using glucocorticoids may additionally have led to reduced reporting to the veterinarian and additional under-reporting of side effects. Furthermore, chronic side effects that may have happened after 31 days of receiving systemic glucocorticoid treatment were likely to be underreported. However, it was considered evaluation of a shorter period after therapy/acute side effects here would reduce the risk that side-effects reported were unrelated to the glucocorticoid therapy administered.

This study was limited by the low power in identifying PUPD due to small number of cases in some categories leading to data sparsity. The study did not analyse data on concurrently administered medications (for example drugs with ulcerogenic or nephrotoxic potential such as NSAIDs) due to the variability in co-therapies used, the range of associated potential side effects and the limited sample size for the PUPD risk factor study undertaken. Nor did the study attempt to extract data on the overall health status of the dogs due to challenges identifying this information consistently within the clinical records of all 3,000 dogs. It is acknowledged these factors in particular could have confounded in part the associations reported and further work would ideally incorporate these variables within the analyses. Clinical indications for the use of systemic glucocorticoids were extracted only for the side effects cases and not for the overall population of dogs treated with systemic glucocorticoids. Knowledge of the prescribed daily doses (mg/kg) could have helped to define the intended therapeutic effect (high “shock” dose, immunosuppression, anti-inflammatory daily, or alternate days) as well as allow evaluation of an association with dose. However, dosage instructions frequently varied within each animal over time (e.g., start one tablet twice a day reduce to one daily then to one every other day) making extraction of these data difficult. Finally, given the study was based on data from 2013 it is possible current available systemic glucocorticoids have changed. Nonetheless, the range of most common systemic glucocorticoids would appear to be broadly comparable, suggesting the study remains relevant (18).

In conclusion, this is the first study to estimate the frequency of side effects and risk factors for PUPD to systemic glucocorticoids occurring with 31 days of treatment in a large general population of dogs attending primary-care practices in UK. The results highlight that combination systemic glucocorticoid therapy and prolonged treatments were associated with increased risk of recording PUPD. Older dogs were at most risk of PUPD. These results can help veterinarians to optimize decision-making when prescribing systemic glucocorticoids for dogs in order to reduce the risks of side effects and to protect the welfare of both dogs and their owners.

## Data Availability Statement

The raw data supporting the conclusions of this article is available on the RVC repository through the following link http://researchonline.rvc.ac.uk/id/eprint/12566/.

## Ethics Statement

Ethical approval was granted by the RVC Ethics and Welfare Committee (URN 2015 1369).

## Author Contributions

DB, DO'N, DE, AW, KM, and LP contributed to the design of the study and the data analysis plan. DO'N and DE organized the database. DE performed the statistical analysis and wrote the manuscript. All authors contributed to the manuscript revision.

## Conflict of Interest

The authors declare that this study received funding from Zoetis. KM and AW were employed by Zoetis and contributed to the design of the study and revision of the manuscript. The research was conducted in the absence of any commercial or financial relationships that could be construed as a potential conflict of interest.

## Abbreviations

BSAVA: British Small Animal Veterinary Association
EPR: electronic patient record
IQR: interquartile range
NOAH: National Office for Animal Heath
OR: odds ratio
PUPD: polyuria and polydipsia
VMD: Veterinary Medicines Directorate.
